# Ruptured Heterotopic Pregnancy Presenting With Hemorrhagic Shock

**DOI:** 10.7759/cureus.88109

**Published:** 2025-07-16

**Authors:** Nuno Prucha Leite, David Costa, Fernando Lino, Mariana S. Santos, François Alves

**Affiliations:** 1 Department of Intensive Care Medicine, Local Health Unit of Entre Douro e Vouga, Santa Maria da Feira, PRT

**Keywords:** hemoperitoneum, hemorrhagic shock, heterotopic pregnancy, intrauterine pregnancy, obstetric emergency, point-of-care ultrasound, ruptured ectopic pregnancy, salpingectomy

## Abstract

Heterotopic pregnancy is an uncommon and potentially life-threatening condition that presents a significant diagnostic challenge, particularly in spontaneous conceptions. We describe the case of a 38-year-old multigravida who presented to the emergency department with acute abdominal pain and signs of hemorrhagic shock. Point-of-care transabdominal ultrasound revealed a moderate volume of intraperitoneal free fluid with echogenic content, raising suspicion for hemoperitoneum or peritonitis, and also demonstrated a viable intrauterine pregnancy at nine weeks’ gestation. Emergency laparoscopy confirmed a ruptured ectopic pregnancy in the right fallopian tube, requiring salpingectomy and peritoneal lavage. Despite initial maternal stabilization, intrauterine fetal death was confirmed on the first postoperative day. This case underscores the need for heightened clinical suspicion in symptomatic pregnant patients, even when an intrauterine pregnancy has been documented and typical risk factors are absent. Timely diagnosis and prompt surgical management remain critical to optimize maternal outcomes.

## Introduction

Heterotopic pregnancy, defined as the simultaneous presence of intrauterine and ectopic gestations, was historically considered exceedingly rare, with an estimated incidence of one in 30,000 spontaneous pregnancies [1]. However, the widespread use of assisted reproductive technologies (ART), such as in vitro fertilization and ovulation induction, has significantly increased its prevalence, with reported rates as high as one in 100 pregnancies among ART users [2,3].

This condition results from the fertilization of multiple ova and subsequent implantation at different sites, typically one within the uterine cavity and another in the fallopian tube. Spontaneous cases are particularly rare and present considerable diagnostic challenges. Heterotopic pregnancy is associated with high maternal morbidity and, in rare instances, mortality. Its clinical presentation is often nonspecific and may mimic other early pregnancy complications, including threatened miscarriage, isolated ectopic pregnancy, corpus luteum hemorrhage, and ovarian torsion. Common symptoms include abdominal pain, vaginal bleeding, and adnexal tenderness. In cases of rupture, patients may exhibit signs of peritoneal irritation and hemodynamic instability.

Diagnosis becomes particularly challenging when a viable intrauterine pregnancy is visualized on ultrasound, as this may create a false sense of reassurance and delay the recognition of a concurrent ectopic gestation [4]. A high index of suspicion is therefore crucial, especially in symptomatic patients, regardless of the prior imaging findings.

This article reviews current concepts in the etiology, risk factors, diagnostic pitfalls, and management of heterotopic pregnancy, illustrated by a rare spontaneous case that underscores the importance of early recognition and timely individualized intervention to prevent life-threatening complications.

## Case presentation

A 38-year-old multigravida presented to the emergency department with acute-onset abdominal pain accompanied by nausea, vomiting, and diarrhea that had persisted for five hours. She denied vaginal bleeding, any history of pelvic inflammatory disease, or previous abdominal surgeries. Her obstetric history included one miscarriage and one cesarean delivery. The current pregnancy was spontaneous and had been routinely monitored; a transvaginal ultrasound performed 14 days prior had confirmed a viable intrauterine pregnancy at six weeks and five days of gestation.

Upon arrival, the patient was in severe distress, exhibiting tachypnea, shallow respirations, central cyanosis, and a peripheral oxygen saturation of 60% on room air. She was tachycardic, pale, diaphoretic, and clinically hypothermic, with unrecordable blood pressure and a Glasgow Coma Scale score of 11 (eye opening to verbal command: 3; verbal response with inappropriate words: 3; motor response localizing to pain: 5). Physical examination revealed signs of peritoneal irritation, including abdominal guarding, rebound and diffuse abdominal tenderness, as well as fecal incontinence.

Initial management included high-flow oxygen therapy via a non-rebreather mask, rapid intravenous fluid resuscitation with crystalloids, analgesia, and preparation for transfusion with cross-matched blood. Laboratory findings revealed metabolic acidosis with hyperlactatemia (lactate: 7.6 mmol/L), hematocrit of 31%, and hemoglobin of 9.5 g/dL (previous baseline: 13.2 g/dL) (Table 1). As part of the initial assessment, point-of-care transabdominal ultrasound demonstrated a moderate volume of free intraperitoneal fluid with echogenic material, raising suspicion for hemoperitoneum or peritonitis. Both ovaries appeared morphologically unremarkable. A fetal heartbeat was identified within a viable intrauterine gestational sac.

**Table 1:** Initial laboratory findings upon emergency department admission in the pregnant patient with hemorrhagic shock The table presents grouped laboratory results obtained upon admission to the emergency department, including complete blood count, coagulation studies, serum biochemistry, arterial blood gas analysis, and microbiological cultures. The findings revealed acute anemia, significant leukocytosis, hyperlactatemia, and metabolic acidosis, with preserved coagulation parameters. No microbiological evidence of infection was found. Overall, this laboratory profile is consistent with hemorrhagic shock and systemic hypoperfusion. * indicates values outside the normal reference range. Reference intervals may vary slightly depending on laboratory standards.

Parameter	Result	Unit	Reference Range
Hemoglobin	9.5*	g/dL	12.0 - 16.0
Hematocrit	31.0*	%	36.0 - 46.0
White blood cells	34.8*	x10⁹/L	4.0 - 11.0
Neutrophils	69.2 / 24.08*	% / x10⁹/L	1.80 - 8.00
Platelets	330	x10⁹/L	150 - 450
International normalized ratio (INR)	1.0	-	-
Prothrombin time (PT)	11.5	s	Control value: 11.4 s
Activated partial thromboplastin time (aPTT)	23.8	s	Control value: 30.6 s
Fibrinogen (Clauss)	217	mg/dL	203 - 472
Sodium	137.0	mmol/L	136 - 145
Potassium	4.0	mmol/L	3.5 - 5.1
Glucose	277*	mg/dL	70 - 105
Urea	26	mg/dL	15 - 40
Creatinine	1.0	mg/dL	0.6 - 1.1
C-reactive protein (CRP)	2.9	mg/L	< 5.0
pH	7.32*	-	7.35 - 7.45
pCO₂	23*	mmHg	35 - 45
Bicarbonate (HCO₃⁻)	11.9*	mmol/L	22 - 28
Lactate	7.6*	mmol/L	< 2.0
Stool culture	Negative	-	-
*E. coli *O157:H7	Negative	-	-
Fecal occult blood	Negative	-	-
Blood culture (1st and 2nd set, aerobic)	Negative	-	-
Blood culture (1st set, anaerobic)	Negative	-	-

Due to persistent hypotension despite fluid resuscitation, vasopressor support was initiated. Broad-spectrum antibiotics were started empirically following the collection of microbiological cultures, given the potential for septic peritonitis.

After multidisciplinary assessment by general surgery and obstetrics/gynecology, the patient underwent emergency exploratory laparoscopy under general anesthesia. Intraoperatively, a massive hemoperitoneum secondary to a ruptured ectopic pregnancy in the right fallopian tube was identified. A right salpingectomy and peritoneal lavage were performed, with approximately 3,700 mL of blood aspirated. The patient remained hemodynamically unstable during the procedure, requiring continued vasopressor support, administration of tranexamic acid, three units of packed red blood cells, and 1 g of fibrinogen.

Given the emergent context and technical limitations of the available equipment, no visual documentation was obtained from the point-of-care ultrasound or intraoperative procedure. Nevertheless, all pertinent findings are comprehensively described in the manuscript.

Follow-up ultrasound on postoperative day one confirmed intrauterine fetal death. The patient was extubated within 24 hours and showed progressive clinical improvement. She was discharged from the intensive care unit on the second postoperative day following resolution of multiple organ dysfunction.

Histopathological examination confirmed a ruptured tubal ectopic pregnancy with hematosalpinx. Six days later, the patient underwent ultrasound-guided suction curettage. She was subsequently followed in surgical and obstetric outpatient clinics, with no further complications reported.

## Discussion

Heterotopic pregnancy is a rare and diagnostically challenging condition, particularly in spontaneous conceptions, and requires a high index of clinical suspicion [4]. Up to 70% of cases are diagnosed between five and eight weeks of gestation [5]. Delayed or missed diagnoses can result in serious complications, including tubal rupture, hypovolemic shock, and maternal death, emphasizing the need for early recognition and timely intervention [6].

In the present case, the patient had no identifiable risk factors typically associated with heterotopic pregnancy, such as pelvic inflammatory disease, previous ectopic pregnancy, tubal surgery, pelvic adhesions, or the use of ART. Her presentation - sudden-onset abdominal pain with guarding, accompanied by nausea, vomiting, and diarrhea, alongside nonspecific ultrasound findings - initially raised concerns for a mixed shock state, potentially combining hypovolemic and septic components.

Transvaginal ultrasonography remains the gold standard for diagnosing heterotopic pregnancy, particularly in high-risk patients during early gestation [7]. However, the identification of a viable intrauterine pregnancy may provide false reassurance and lead to the erroneous exclusion of a concurrent ectopic gestation. In this case, point-of-care ultrasound in the emergency department revealed significant intraperitoneal free fluid, prompting urgent surgical exploration and intraoperative confirmation of tubal rupture.

The presence of free intraperitoneal fluid in a pregnant patient, particularly when accompanied by hemodynamic instability or signs of peritoneal irritation, should raise immediate suspicion for heterotopic pregnancy, even when a live intrauterine pregnancy has already been documented.

Management is primarily guided by the patient’s hemodynamic status and the timing of presentation. In unstable patients or cases of confirmed rupture, prompt surgical intervention (either by laparoscopy or laparotomy) is essential to control hemorrhage and reduce maternal morbidity and mortality. When feasible, efforts may be made to preserve the intrauterine pregnancy. Nevertheless, surgical management carries inherent risks, including miscarriage rates reportedly as high as 15% [2,7-9].

In this case, emergency laparoscopy and right salpingectomy were performed due to ongoing hemodynamic instability. The patient’s recovery required critical care support, including vasopressors and blood product transfusions. Effective multidisciplinary coordination, from the emergency department to surgical and critical care teams, was crucial to achieving a favorable maternal outcome.

This case is particularly notable for the coexistence of hemoperitoneum, signs of peritoneal irritation, declining hemoglobin levels, and hemodynamic collapse in a patient with a viable intrauterine pregnancy and no apparent risk factors. It highlights the importance of maintaining heterotopic pregnancy in the differential diagnosis of any pregnant patient presenting with abdominal pain and shock, regardless of prior imaging findings or clinical history.

## Conclusions

This case illustrates a rare but life-threatening presentation of hemorrhagic shock caused by a ruptured heterotopic pregnancy - an obstetric emergency that can easily be overlooked, especially in the presence of a confirmed intrauterine gestation. It underscores the importance of maintaining heterotopic pregnancy in the differential diagnosis of symptomatic pregnant patients, particularly those presenting with abdominal pain and hemodynamic instability. Early recognition and timely surgical intervention are crucial to improving maternal outcomes and preventing severe complications.
